# Localization of uPAR and MMP-9 in lipid rafts is critical for migration, invasion and angiogenesis in human breast cancer cells

**DOI:** 10.1186/1471-2407-10-647

**Published:** 2010-11-24

**Authors:** Hari Raghu, Prasanna Kumar Sodadasu, Rama Rao Malla, Christopher S Gondi, Norman Estes, Jasti S Rao

**Affiliations:** 1 Department of Cancer Biology and Pharmacology, University of Illinois College of Medicine at Peoria, One Illini Drive, Peoria, IL 61605, USA; 2 Department of Surgery, University of Illinois College of Medicine at Peoria, One Illini Drive, Peoria, IL 61605, USA; 3 Department of Neurosurgery, University of Illinois College of Medicine at Peoria, One Illini Drive, Peoria, IL 61605, USA

## Abstract

**Background:**

uPAR and MMP-9, which play critical roles in tumor cell invasion, migration and angiogenesis, have been shown to be associated with lipid rafts.

**Methods:**

To investigate whether cholesterol could regulate uPAR and MMP-9 in breast carcinoma, we used MβCD (methyl beta cyclodextrin, which extracts cholesterol from lipid rafts) to disrupt lipid rafts and studied its effect on breast cancer cell migration, invasion, angiogenesis and signaling.

**Results:**

Morphological evidence showed the association of uPAR with lipid rafts in breast carcinoma cells. MβCD treatment significantly reduced the colocalization of uPAR and MMP-9 with lipid raft markers and also significantly reduced uPAR and MMP-9 at both the protein and mRNA levels. Spheroid migration and invasion assays showed inhibition of breast carcinoma cell migration and invasion after MβCD treatment. *In vitro *angiogenesis studies showed a significant decrease in the angiogenic potential of cells pretreated with MβCD. MβCD treatment significantly reduced the levels of MMP-9 and uPAR in raft fractions of MDA-MB-231 and ZR 751 cells. Phosphorylated forms of Src, FAK, Cav, Akt and ERK were significantly inhibited upon MβCD treatment. Increased levels of soluble uPAR were observed upon MβCD treatment. Cholesterol supplementation restored uPAR expression to basal levels in breast carcinoma cell lines. Increased colocalization of uPAR with the lysosomal marker LAMP1 was observed in MβCD-treated cells when compared with untreated cells.

**Conclusion:**

Taken together, our results suggest that cholesterol levels in lipid rafts are critical for the migration, invasion, and angiogenesis of breast carcinoma cells and could be a critical regulatory factor in these cancer cell processes mediated by uPAR and MMP-9.

## Background

Lipid rafts are detergent-insoluble, cholesterol-rich microdomains and have been attributed to several cellular functions. Lipid rafts play critical roles in the regulation of several membrane receptors, apoptosis, cell adhesion, and protein sorting during endocytosis and exocytosis [1,2]. Lipid rafts have also been implicated in invasion, viral entry and egress, cholesterol metabolism, endocytosis, etc. but many of their functions are still unknown [3]. Lipid rafts are known to be abundant in signaling molecules and to regulate signal transduction by activating or suppressing phosphorylation cascades related to growth, survival and many other physiological processes [4]. They are also known to function as molecular platforms that organize appropriate molecules for specific signaling pathways [5].

uPAR, a glycosylphosphatidylinositol-anchored membrane protein with multiple functions in extracellular proteolysis, cell adhesion, cell migration and cell proliferation, is found in both the lipid rafts and in more fluid regions of the plasma membrane [6]. Cunningham and colleagues [7] have shown that dimerized uPAR partitions preferentially to detergent-resistant lipid rafts. uPAR seems to associate with L-selectin in human neutrophil lipid rafts but not with the complement receptor CR3 [8]. Matrix metalloproteinases comprise a family of zinc-binding endopeptidases that are capable of degrading ECM components, including collagen and proteoglycan [9]. MMP-2 and MMP-9 play very critical roles in cancer, infectious disease, wound healing, inflammation and many vascular diseases [10-13]. Zhang et al. [14] have shown that MMP-9 associates with lipid rafts in highly metastatic sublines from Lewis Lung carcinoma.

Cholesterol is a important component of lipid rafts and is known to play a important role in maintaining membrane integrity, trafficking, signal transduction and fluidity [15-17]. Cholesterol depletion results in the disorganization of lipid raft microdomains and also the dissociation of proteins [18] that are bound to the lipid raft. Cholesterol depleting agents, such as filipin, nystatin or methyl beta cyclodextrin (MβCD), remove cholesterol and cause disruption of lipid rafts within a short period of time. MβCD is a strictly surface acting agent, can selectively and rapidly remove cholesterol from the plasma membrane in preference to other membrane lipids, and has been widely used in studying the effects of cholesterol depletion on lipid raft assembly. Moreover, cholesterol accumulation is known to be associated in many tumors, including prostate cancer and oral cancer [19,20], and dysregulated in lung and breast cancers [21,22].

As uPAR and MMP-9 are known to be associated with cholesterol-enriched lipid rafts, the present study investigated the effects of cholesterol depletion-mediated lipid raft disruption by MβCD treatment on uPAR and MMP-9 in breast carcinoma cells. Overall, in our study, we demonstrated that MβCD treatment inhibited raft-associated uPAR and MMP-9 activity in MDA-MB-231 and ZR 751 cells. MβCD treatment down regulated the phosphorylation of Src, FAK, Cav, ERK, and Akt and targeted uPAR to the lysosomal pathway in breast carcinoma cells. Our results show the implications of cholesterol depletion in uPAR and MMP-9-related cancer cell functions and provide a biological basis for targeting lipid rafts in future breast cancer therapy and treatment.

## Methods

### Cells, antibodies and reagents

MDA MB231, ZR 751, HMEC cells were cultured according to methods established in our laboratory (24). MβCD, nystatin and NAC were purchased from Sigma (St. Louis, MO). Anti-Src-pY416, anti-Src-pY527, and pAkt were purchased from Cell Signaling (Danvers, MA). Antibodies for the phosphorylated and total forms of ERK, FAK, PI3K, Akt and Cav were purchased from Santa Cruz Biotechnology (Santa Cruz, CA). Antibodies against MMP-9, flotillin and CD71 were purchased from Santa Cruz Biotechnology (Santa Cruz, CA). The uPAR antibody was purchased from R&D Systems (Minneapolis, MN). Secondary antibodies were also purchased from Santa Cruz Biotechnology (Santa Cruz, CA). Alexa Fluor antibodies and Vybrant lipid raft labeling kit were purchased from Invitrogen (Carlsbad, CA). Caveolae/raft isolation kit was purchased from Sigma (St. Louis, MO).

### Cytotoxicity assays

Target cells were incubated with Dulbecco's modified Eagle medium (DMEM) containing different concentrations of MβCD for 1, 4, 24 and 48 hrs. Supernatants were collected and assessed for cellular toxicity using a LDH cytotoxicity assay kit (Promega, Madison, WI) in accordance with the manufacturer's instructions.

### Determination of cellular cholesterol by Amplex Red cholesterol assay

MDA-MB-231 and ZR 751 cells were washed twice with phosphate-buffered saline (PBS) and then incubated at 37°C for 1 hr with different concentrations of MβCD in DMEM. After two washes with PBS, cells were harvested for cholesterol measurement assays as per the manufacturer's instructions (Molecular Probes Inc, Eugene, OR).

### Colocalization studies

Confluent MDA-MB-231 and ZR 751 cells grown in eight-well chamber slides were left untreated or pretreated with different concentrations of MβCD for one hour at 37°C, washed and labeled for lipid raft marker flotiilin or with GM1 (lipid raft labeling kit, Molecular Probes) as per the manufacturer's instructions. The cells were washed, fixed with 2% paraformaldehyde, permeabilized in 0.5% Triton X-100, and stained with primary antibodies against uPAR, MMP-9, pFAK, pCav, pPI3K, and GM1 overnight at 4°C. The cells were then washed, incubated with appropriate secondary antibodies for 1 hr at room temperature, washed, mounted with anti-fading agent containing DAPI, and examined under a confocal fluorescence microscope. For colocalization studies of uPAR and LAMP-1, MDA-MB-231 cells grown in eight-well chamber slides were left untreated or treated with 7.5 mM MβCD, fixed with 2% paraformaldehyde at indicated time points (1, 8 and 24 hrs), and incubated with primary antibodies against uPAR and LAMP-1. The cells were then washed and further incubated with appropriate secondary Alexa Fluor antibodies, and examined under a Olympus fluoview confocal microscope.

### Spheroid migration assay

MDA-MB-231 and ZR 751 cells (1.5 × 10^4^) were suspended in the appropriate medium, seeded onto 0.5% agar-coated plates, and cultured until spheroids formed. Intact tumor spheroids were selected and transferred to six-well plates. The spheroids were either left untreated or treated with different concentrations of MβCD and followed by incubation for 48 hrs. Then, the spheroids with migrated cells were fixed with 10% buffered formalin in PBS and stained using crystal violet staining solution. The spheroids were observed under a normal light microscope and photographed at 20× and 40× magnification using the DP controller imaging software.

### Fibrin and gelatin zymography

Conditioned medium was collected from MDA-MB-231 and ZR 751 control cells and cells that were treated with different concentrations of MβCD. MDA MB231 and ZR 751 cells pretreated with ruPA (10 ng/μl) and rMMP-9 (2.5 ng/μl) were used as positive controls. The conditioned media was subjected to fibrin and gelatin zymography as described by Gondi et al. [23]. MMP-9 activity was quantified as arbitrary units and compared with controls.

### Preparation of RNA

Isolation of total RNA from treated and untreated cells was carried out using the TRIzol reagent (Invitrogen, Carlsbad, CA) according to the manufacturer's instructions.

### Reverse transcription-PCR

Total RNA was isolated from cells in all treatment conditions using the TRIzol reagent as per the standard protocol. Total RNA was treated with DNAse I (Invitrogen, Carlsbad, CA) to remove contaminating genomic DNA. PCR analysis was done using the one-step reverse transcription-PCR kit (Invitrogen, Carlsbad, CA). Glyceraldehyde 3-phosphate dehydrogenase (GAPDH) was used as an internal control. The following primers were used:

**uPAR**: 5'-GAAGGAGAGAAGACGTGCAG-3' sense

5'-GATCCAGCCAGGGCAGAG-3' antisense

**MMP-9**: 5'-TGGACGATGCCTGCAACGTG-3' sense

5'-TGCCTTTGGACACGCACGAC-3' antisense

**GAPDH**:5'-CGGAGTCAACGGATTTGGTCGTAT-3'sense 5'-AGCCTTCTCCATGGTGGTGAAGAC 3'antisense

The PCR conditions were as follows: 95°C for 5 min, followed by 30 cycles of 95°C for 1 min, and annealing temperature set according to the AT and GC content of the primers.

### Western blotting

MDA-MB-231 and ZR 751 cells were either left untreated or pretreated with different concentrations of MβCD for 1 hr at 37°C. Total cell lysates were prepared and resolved on SDS-polyacrylamide gels, transferred onto nitrocellulose paper, and immunoblotted with appropriate primary antibodies.

### uPAR immunoassay

Untreated MDA-MB-231 and ZR 751 cells and cells pretreated with different concentrations of MβCD for 1 hr at 37°C were allowed to incubate with serum-free medium for 24 and 48 hrs. Conditioned medium collected from the treated and untreated cells were subjected to uPAR immunoassay as per the manufacturer's instructions (R&D Systems, Minnepolis, MN).

### *In vitro *angiogenesis assay

MDA-MB-231 cells (2 × 10^4^) and ZR 751 cells were seeded in 100-mm plates and either left untreated or treated with different concentrations of MβCD for 1 hr at 37°C. After treatment, the medium was removed; washed and serum-free medium was added. Conditioned medium was collected after 24 hrs. Human microvascular endothelial cells (HMEC) were cultured in the conditioned medium for 24 hrs. After the incubation period, the medium was removed, and the cells were stained with Hema-3 stain (Fisher Diagnostics, Fisher Scientific Company, Middletown, VA) and examined under a microscope. Image Pro software (Media Cybernetics, Silver Spring, MD) was used for quantification of angiogenesis. The degree of angiogenesis was measured by the following method: number of branch points and the total number of branches per point, with the product indicating the degree of angiogenesis.

### Matrigel invasion assay

MDA-MB 231 cells (1 × 10^6^) were left untreated or pretreated with different concentrations of MßCD for 1 h at 37°C, washed and allowed to invade through matrigel - coated transwell inserts (8 μM pores) for 24 hrs. Cells which invaded through the Matrigel coated inserts were stained, counted and photographed under an Olympus light microscope at 20× magnification. Quantitative estimation of invasion was done as per the method previously established in our laboratory [24].

### Biochemical isolation of detergent-resistant domain association and cholesterol depletion

MDA-MB-231 and ZR 751 cells (1-2 × 10^7^/25-cm^2 ^culture flasks) were serum starved for 14 to 16 hrs, and cells were left untreated, pretreated with 7.5 mM MβCD or 10 mM MβCD for 1 hr at 37°C to allow for cholesterol depletion (60%). Lipid raft fractions were isolated using the Raft Isolation Kit (Sigma, Inc., St.Louis, MO) Rafts were also isolated using the procedure of Raghu et al. [3]. The whole procedure was performed at 0-4°C. DRM and soluble fractions were collected and subjected to electrophoresis and Western blotting. Fractions 3, 4, and 5 were pooled and marked as raft fractions and fractions 6, 7, and 8 were labeled as non-raft fractions. Raft fractions and non-raft fractions were characterized based on the specificity to caveolin and transferrin in MDA-MB-231 cells and flotillin and transferrin in ZR 751 cells.

### Cholesterol depletion and supplementation experiments

Cholesterol depletion was done by treating cells with different concentrations of MβCD for 1 hr at 37°C followed by washing and incubation with serum-free DMEM for the indicated time periods. For cholesterol supplementation, the cells were treated initially with 10 mM MβCD, and then the cells were incubated for different time periods with or without the cholesterol MβCD complexes. At the end of each indicated time period, the cells were washed and incubated with serum-free medium, and the lysates were collected for western blot analysis.

### Statistical analysis

All statistical analyses were carried out using Excel software. The significance of the differences was determined using an independent samples t-test. A *p *value of less than 0.05 was regarded as statistically significant.

### RESULTS: uPAR colocalizes with GM1 and flotillin, two lipid raft markers, in MDA-MB-231 and ZR 751 cells

**Figure 1:**
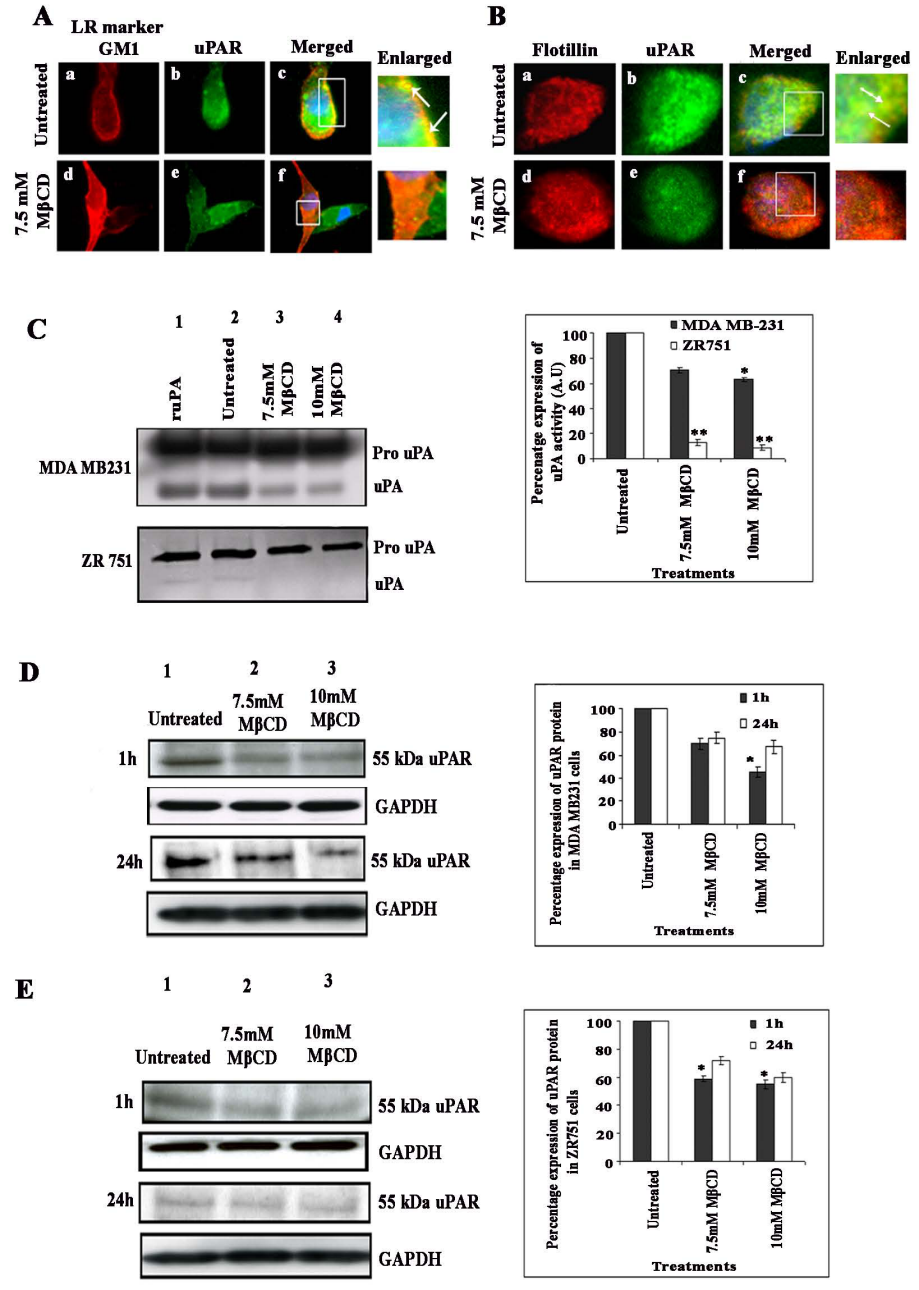
**uPAR colocalizes with lipid rafts in MDA-MB-231 and ZR 751 cells**. (A) and (B) MDA-MB-231 and ZR 751 cells grown in 8-well chamber slides were serum starved for 12-16 hrs, either left untreated or pretreated with MβCD, washed, labeled for the lipid raft marker GM1, fixed, permeabilized and blocked with 5% BSA for 1 hr. Then, cells were washed, labeled with anti-uPAR antibody for 1 hr at room temperature, washed, and stained for 1 hr with Alexa Fluor 488 (uPAR) and Alexa Fluor 594 (GM1) conjugated secondary antibodies at room temperature. Panels a to c: untreated cells; panels d to f: cells pretreated with 7.5 mM MβCD. The insets in panel's c and f show an enlarged view of the colocalization. Arrows indicate colocalization of uPAR with GM1 in MDA-MB-231 cells and uPAR and flotillin in ZR 751 cells before and after treatment with MβCD. Scale bars: 10 μM. (C) Inhibition of uPA activity by fibrin zymography. MDA-MB-231 and ZR 751 cells were left untreated or pretreated with different concentrations of MβCD, treated with ruPA (positive control) (10 ng/μl) washed and serum-free medium was added. After 24 hrs, conditioned medium was collected, and uPA activity was measured by fibrin zymography in MDA-MB-231 and ZR 751 cells. Densitometric analysis was done using the ImageJ software (**p < 0.001). (D) Western blot analysis of uPAR protein expression at 1 hr and 24 hr time points in cell lysates from MDA-MB-231 (D) and ZR 751 (E) cells either left untreated or pretreated with different concentrations of MβCD. Western blot analysis was performed with an antibody specific for uPAR. GAPDH was simultaneously immunodetected to verify equal loading of cell lysates. Densitometric analysis was done for uPAR expression (*p < 0.01). The result provided is a representative experiment repeated 3 times with concordant results.

As previously reported [6,7], uPAR, a GPI-anchored receptor protein, is a lipid raft-associated protein. In Figure 1A, GM1 staining, which is an LR specific marker, was observed predominantly in the cell periphery of unstimulated MDA-MB-231 cells (Figure 1A, panel a). Also, uPAR staining was seen more near the plasma membrane (Figure 1A, panel b). A very clear margin of colocalization of uPAR the with LR marker GM1 was observed near the plasma membrane of MDA-MB-231 cells (Figure 1A, panel c). Colocalization studies were done with GM1 at 1 h and 24 h time points after 1 h treatment with MβCD. In cells pretreated with 7.5 mM MβCD, we found disrupted GM1 spots in the cells (Figure 1A, panel d). In addition, we observed inhibition of uPAR staining at the plasma membrane (Figure 1A, panel e). Moreover, we did not observe colocalization of GM1 with uPAR in MβCD-treated cells (Figure 1A, panel f). Morphological analysis revealed the association of uPAR with another lipid raft marker, flotillin, in ZR 751 cells (Figure 1B, panels a to c). In cells pretreated with MβCD, we observed significant inhibition of the colocalization of flotillin with uPAR in ZR 751 cells (Figure 1B, panels d to f).

### Inhibition of uPA and uPAR protein expression by the lipid raft disrupting agent MβCD in MDA-MB-231 and ZR 751 cells

In untreated cells, we observed increased activity of uPA in MDA-MB-231 and ZR 751 cells as assessed by fibrin zymography (Figure 1C, lane 2 top panel (MDA MB231), bottom panel, lane 2(ZR 751). In cells treated with MβCD, inhibition of uPA activity was observed at 24 h time point (Figure 1C top panel lane 3 & 4 (MDA MB 231) and bottom panel lane 3 & 4 (ZR 751). Conditioned medium from cells treated with ruPA (10 ng/μl) was taken as a positive control (Figure 1C lane 1, top and bottom panel). Lysates from two time points namely at 1 hr and 24 hrs showed significant decreases in uPAR protein levels in LR-disrupted cells when compared with untreated controls. The decrease in expression levels was also dose-dependent; 10 mM MβCD treatment resulted in 40% decrease in expression levels while 7.5 mM MβCD treatment resulted in 30% decrease in expression levels in MDA-MB-231 cells (Figure 1D, lanes 2 and 3). Equal loading of total lysates was confirmed with GAPDH. In ER-positive breast cancer cells, we observed a similar pattern of uPAR expression in unstimulated cells. In cells treated with different concentrations of MβCD, we observed a 30-35% reduction in uPAR protein levels (Figure 1E, lanes 2 and 3). uPAR expression in ZR 751 cells was observed for up to 24 hrs (Figure 1E, lanes 2 and 3). These results suggest that lipid raft disruption by plasma membrane cholesterol depletion plays an active role in uPA and uPAR expression levels in both MDA-MB-231 and ZR 751 cells.

### MMP-9 colocalizes with the lipid raft marker GM1 in MDA-MB-231 and with flotillin in ZR 751 cells

**Figure 2:**
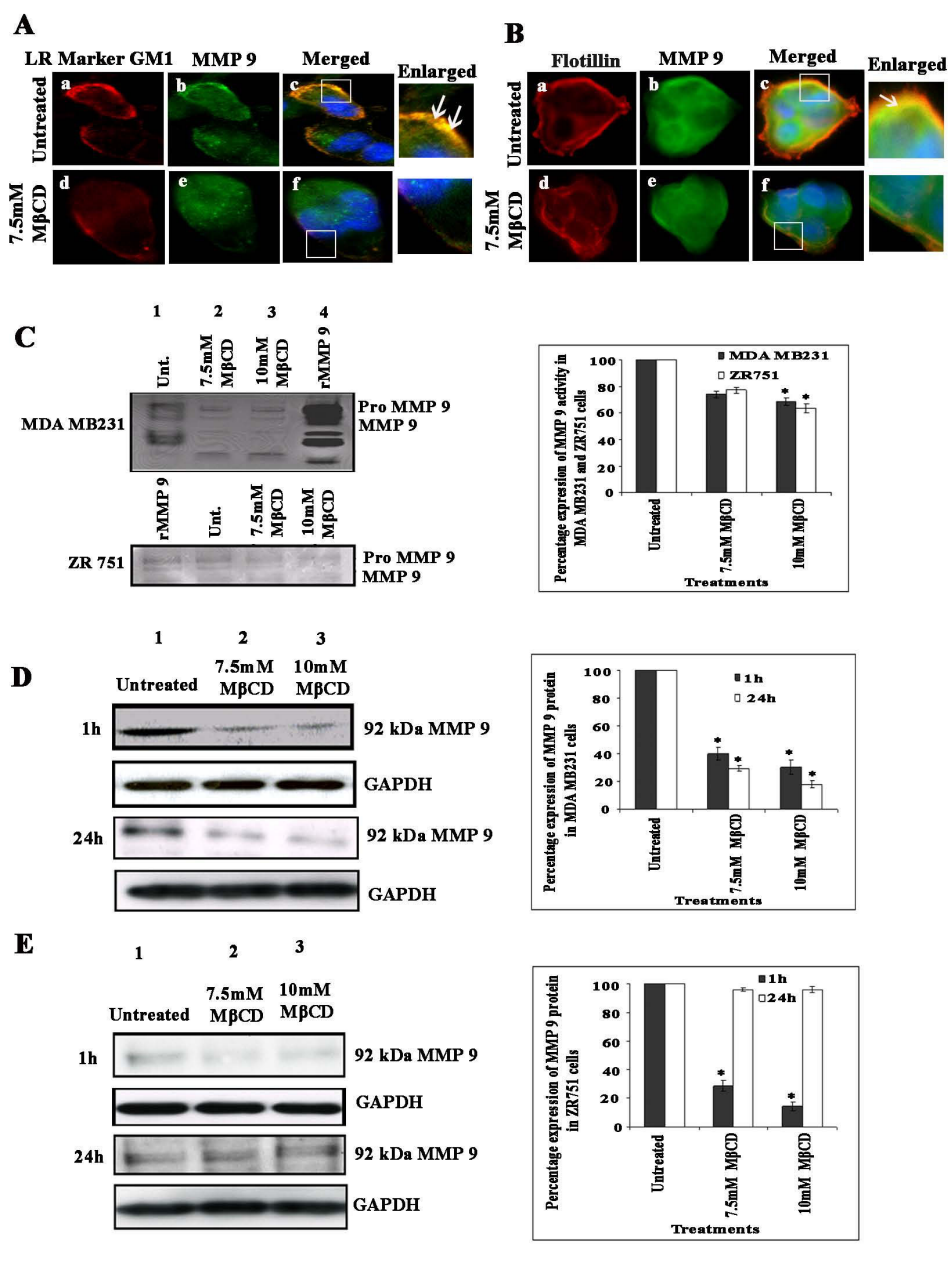
**MMP-9 colocalizes with lipid rafts in MDA MB 231 and ZR 751 cells**. (A) and (B) MDA-MB-231 and ZR 751 cells grown in 8-well chamber slides were serum starved for 12-16 hrs, either left untreated or pretreated with different concentrations of MβCD, washed, labeled for the lipid raft marker GM1 using vibrant lipid raft labeling kit, fixed, permeabilized and blocked with 5% BSA for 1 hr. Then, cells were washed, labeled with anti-MMP-9 antibody for 1 hr at room temperature, washed and stained for 1 hr with Alexa Fluor 488 (MMP-9) conjugated secondary antibody at room temperature. Panels a to c: untreated cells; panels d to f: cells pretreated with 7.5 mM MβCD. The insets in panel's c to f showed an enlarged view of the colocalization. Arrows indicate colocalization of MMP-9 with GM1 in MDA-MB-231 cells and with flotillin in ZR 751 cells. Scale bars: 10 μM. (C) Inhibition of MMP-9 activity as shown by gelatin zymography in MDA-MB-231 and ZR 751 cells left untreated, pretreated with different concentrations of MBCD or treated with rMMP-9 (2.5 ng/μl). Densitometric analysis was done using the ImageJ software (**p < 0.001). (D) Western blot analysis of MMP-9 protein expression at 1 hr and 24 hr time points in cell lysates from MDA-MB-231 (D) and ZR 751 (E) cells left untreated or pretreated with different concentrations of MβCD. Western blot analysis was performed with an antibody specific for MMP-9. GAPDH was simultaneously immunodetected to verify equal loading of the lysates. Densitometric analysis was done for MMP-9 expression (*p < 0.01, **p < 0.001). The result provided is a representative experiment repeated three times with concordant results.

Previous studies have shown that MMP-9 is associated with lipid rafts (14). To ascertain if such an association exists in breast carcinoma cells, we used untreated and MβCD-treated breast carcinoma cells and checked for colocalization of MMP-9 with GM1 in MDA-MB-231 cells and with flotillin in ZR 751 cells. As expected, we observed lipid raft staining at the cell periphery in MDA-MB-231 cells (Figure 2A, panel a), and MMP-9 staining was more concentrated at the leading edge of the cells (Figure 2A, panel b). In the merged image, we observed colocalization of MMP-9 with the lipid raft marker GM1 at the leading edge and at the margin of the cell, suggesting that MMP-9 associates with lipid rafts at the leading edge and in the periphery of the cells. When the cells were pretreated with 7.5 mM MβCD for 1 hr at 37°C, we did not observe colocalization of MMP-9 and GM1 (Figure 2A, panels d to f). In ZR 751 cells, we observed colocalization of MMP-9 with the lipid raft marker flotillin in untreated cells (Figure 2B, panels a to c) while in cells treated with 7.5 mM MβCD, we observed significant inhibition of the colocalization of MMP-9 with flotillin (Figure 2B, panels d to f). These results suggest that MMP-9 colocalizes with lipid rafts, and lipid raft disruption by cholesterol depletion inhibits this association.

### Lipid raft disruption decreases MMP-9 activity and protein expression in MDA-MB-231 cells and ZR 751 cells

Morphological studies have shown that MMP-9, a zinc endopeptidase, associates with lipid rafts at the periphery and at the leading edge of the cells. To ascertain if lipid raft disruption by cholesterol depletion inhibits MMP-9 activity, we determined MMP-9 activity using gelatin zymography. Consistent with our previous results [24], we observed increased MMP-9 activity in conditioned medium at the 24 hr time point in untreated cells (Figure 2C, lane 1 top panel (MDA MB231) and Figure 2C lane 2 bottom panel (ZR 751). However, in the MβCD-treated cells, there was 25-35% decrease in MMP-9 activity (Figure 2C, lanes 2 and 3 (MDA MB 231 and Figure 2C lane 3, 4 (ZR 751). As a positive control the cells were treated with rMMP-9 (2.5 ng/μl) (Figure 2C lane 4 top panel (MDA MB231) and Figure 2C lane 1 bottom panel (ZR 751). To further substantiate our results, we wanted to determine if lipid raft disruption also affected the MMP-9 protein expression levels in MDA-MB-231 and in ZR 751 cells. MMP-9 levels in the total cell lysates were measured using western blotting. As shown previously, we observed a higher level of MMP-9 expression in untreated cells (Figures 2D-E, lane 1) as compared with MDA-MB-231 and ZR 751 cells pretreated with different concentrations of MβCD (Figure 2D, lanes 2 and 3). The MMP-9 expression levels in MDA-MB-231 cells showed a decrease in MMP-9 protein levels up to 24 hrs in MβCD-treated cells. Equal loading of lysates is demonstrated by GAPDH expression (Figure 2D, top and bottom panel). The MMP-9 expression levels in ZR 751 cells showed a decrease in MMP-9 protein levels up to 1 hr in MβCD-treated cells. At the 24 hr time point, we did not observe significant changes in MMP-9 levels in untreated and lipid raft-disrupted ZR 751 cells (Figure 2E, lanes 2 and 3). These results show that in both ER-positive and ER-negative cell lines, lipid rafts are critical for uPAR and MMP-9 expression and activity. The differences in MMP-9 protein expression levels in MDA-MB-231 and ZR 751 cells demonstrate the cell type variation in MMP-9 levels mediated by lipid raft disruption.

### Biochemical isolation of lipid rafts demonstrates uPAR and MMP-9 in lipid raft fractions of MDA-MB-231 and ZR 751 cells

**Figure 3:**
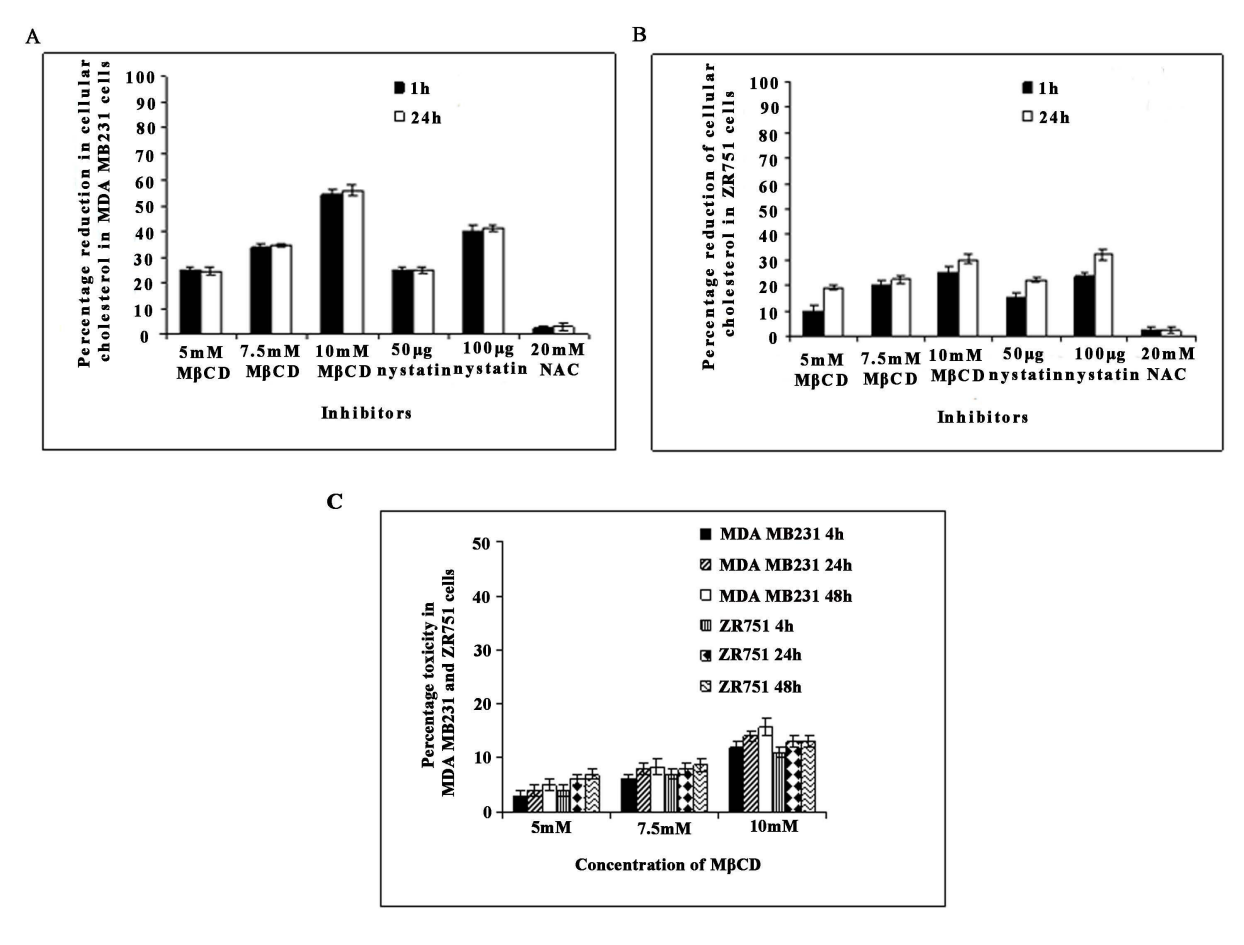
**Effect of MβCD and nystatin on MDA-MB-231 and ZR 751 cells**. **(A) and (B)**. Cholesterol depletion/chelation by MβCD and nystatin. MDA-MB-231 and ZR 751 cells were treated with different concentrations of MβCD and nystatin for 1 hr, and cholesterol levels were measured at 1 and 24 hrs in (A) MDA-MB-231 and (B) ZR 751 cells. The percentage reduction in cholesterol upon drug treatment was calculated with respect to total cholesterol in the untreated cells, which is taken as 100%. Each reaction was performed in duplicate and each point represents the mean ± S.D. of three independent experiments. (C) MDAMB 231 and ZR 751 cells were incubated with different concentrations of MβCD for 1 h, 4 h, 24 h and 48 h. Supernatants were collected and assessed for cellular toxicity by using a lactate dehydrogenase assay kit. The data is represented as percentage cytoxicity with reference to untreated cells.

To determine whether treatment of MDA-MB-231 and ZR 751 cells with various concentrations of MβCD and nystatin efficiently extracted cholesterol and to check whether the cholesterol levels remained depleted even after 24 hrs, we assayed cellular cholesterol levels. As shown in Figures 3A and 3B, after treatment with non-toxic doses of the drugs, cellular cholesterol levels decreased in a dose-dependent manner at both the 1 hr and 24 hr time points in both of the cell lines tested. With 50 and 100 μg concentrations of nystatin, we observed a 20-30% reduction in ZR 751 cells at 1 hr and 24 hrs; in MDA-MB-231 cells, we observed a 25-40% reduction in cellular cholesterol levels (Figures 3A-B). With 5 mM, 7.5 mM and 10 mM MβCD, we found 20-30% reduction in cellular cholesterol levels in ZR 751 cells and 25-55% reduction in MDA-MB-231 cells (Figure 3A). In contrast, we did not observe a significant change in cellular cholesterol levels when cells were treated with 20 mM NAC (n-acetyl cysteine (a known antioxidant) for 1 hr at 37°C (Figures 3A-B). To further confirm that concentration of MβCD used is nontoxic to the cells, we did the cellular cytotoxicity assay using the Lactate dehydrogenase assay kit. Results showed that both MDA MB231 and ZR 751 cells tolerated 5 mM, 7.5 mM and 10 mM concentrations of MβCD upto 48 h time points and the concentrations used were nontoxic to the cells. (Figure 3C). These results demonstrate that both MβCD and nystatin were efficient in depleting or chelating cholesterol from the lipid rafts of MDA-MB-231 and ZR 751 cells under our testing conditions.

**Figure 4:**
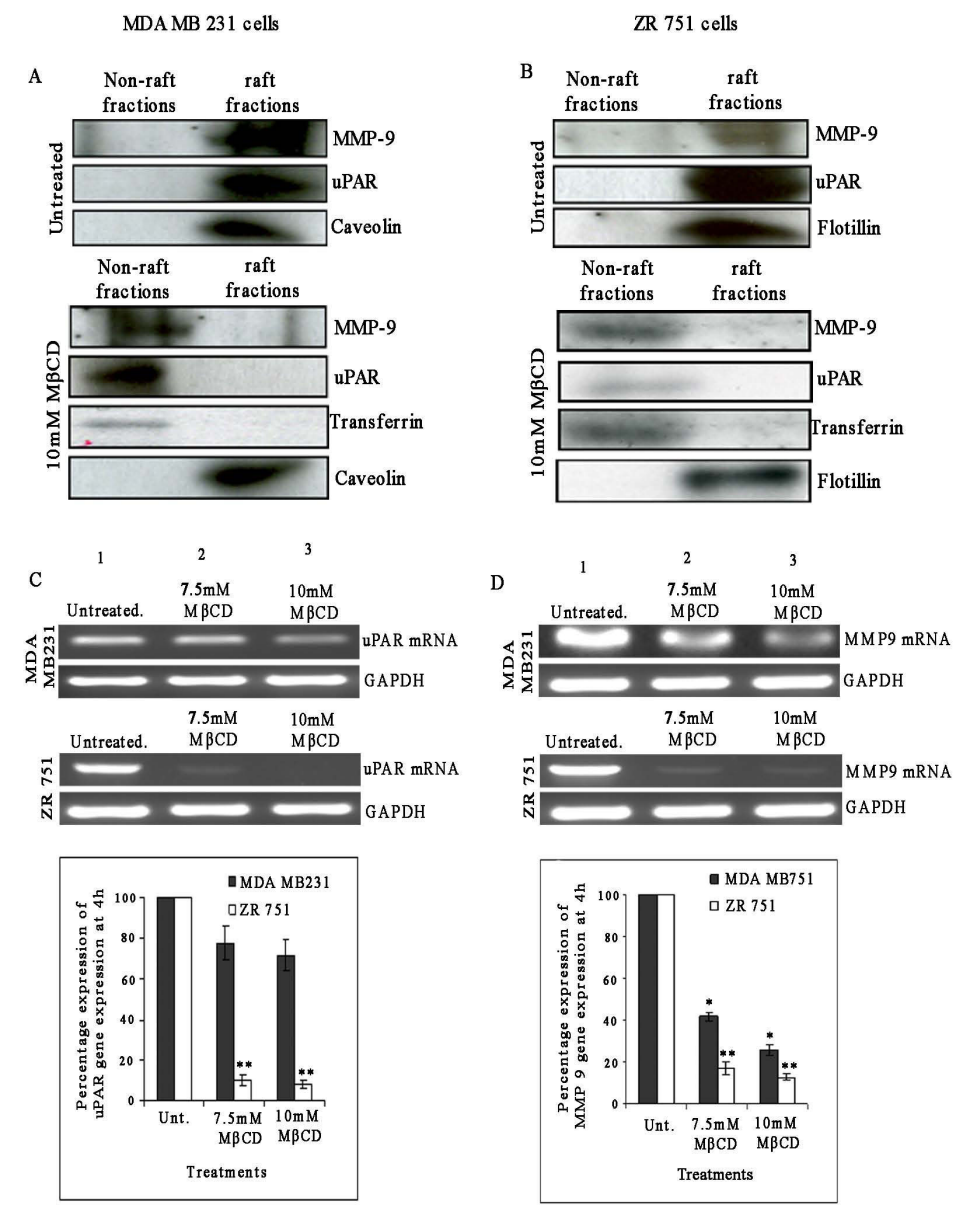
**Lipid raft disruption reduces uPAR and MMP-9 activity in lipid raft fractions of MDA-MB-231 and ZR 751 cells**. (A) & (B) Characterization of lipid raft fractions. (A) MDA-MB-231 and (B) ZR 751 cells were left untreated or pretreated with 10 mM MβCD for one hour. Cells were washed, lysed, and raft and non-raft fractions were collected as described earlier. Fractions were characterized as raft fractions or non-raft fractions and western blotted for caveolin or flotillin (raft marker) and transferrin (non-raft marker). Fractions 3 to 5 were pooled as raft fractions, and pooled fractions 6 to 8 were taken as non-raft fractions. (C) & (D) Effect of lipid raft disruption on uPAR and MMP-9 gene expression in breast carcinoma cells. (C) MDA-MB-231 and (D) ZR 751 cells were left untreated or pretreated with different concentrations of MβCD for one hour, washed, complete medium added, and total RNA isolated at 8 h using the TRIzol method. 50 ng of DNAse-treated RNA/μL were subjected to semi quantitative RT-PCR with primers specific for uPAR and MMP-9. Expression of GAPDH was used to verify equal loading of cDNA. (D) Densitometric analysis of uPAR and MMP-9 expression at the mRNA level (mean ± SE, n = 3) (*p < 0.001, **p < 0.0001).

As shown previously in Figure 1C and Figure 2C, when lipid rafts were disrupted by depletion of plasma membrane cholesterol levels in MDA-MB-231 and ZR 751 cells, we observed decreased uPA and MMP-9 activity in the conditioned medium at the 24 hr time point. In addition, we found significant decreases in uPAR and MMP-9 protein levels in the LR-disrupted lysates as compared with untreated lysates. Next, we determined whether uPAR and MMP-9 are associated with LR microdomains by biochemical isolation of lipid raft fractions from both cell types. We carried out biochemical isolation of lipid rafts using the detergent method [25]. Fractions 3, 4 and 5, which were positive for the lipid raft marker caveolin (Figure 3A, left upper panel) and negative for transferrin (Figure 4A, left bottom panel), were pooled and labeled as lipid raft fractions. Fractions 6, 7 and 8, which were positive for transferrin, a non-raft marker, (Figure 4A, right bottom panel) and negative for caveolin (Figure 4A, right upper panel), were pooled and labeled as non-raft fractions. uPAR and MMP-9 in the pooled raft fractions (3 to 5) and non-raft fractions (6 to 8) were examined by immunoprecipitation assays. Higher levels of uPAR and MMP-9 were observed in raft fractions of both MDA-MB-231 and ZR 751 cells (Figures 4A-B) than in the non-raft fractions. MβCD treatment significantly decreased the association of uPAR and MMP-9 with the raft fractions, resulting in little or no activity similar to non-raft fractions. These results suggest that both uPAR and MMP-9 associate with lipid rafts in MDA-MB-231 and ZR 751 cells.

### Lipid raft disruption by MβCD inhibits uPAR and MMP-9 mRNA gene expression

As previously shown in Figure 1C-E and in Figure 2C-E, lipid raft disruption downregulated uPAR, MMP-9 and uPA activity and protein expression in both MDA-MB-231 and ZR 751 cells. As a consequence, we assessed whether lipid raft disruption affects gene expression in breast carcinoma cells. MDA-MB-231 and ZR 751 cells were left untreated or pretreated with different concentrations of MβCD for one hour at 37°C, and total RNA was extracted at 4, 8 and 24 hrs. uPAR mRNA and MMP-9 mRNA levels were measured by semi-quantitative RT-PCR. As shown in Figures 4C-D, we observed significant inhibition of uPAR mRNA in both MDA-MB-231 and ZR 751 cells at early time points up to 8 hrs after treatment. mRNA analysis at the 24 hr time point showed no changes in uPAR mRNA or MMP-9 mRNA in LR-disrupted cells (data not shown). These results further strengthened our previous findings, and the downregulation of uPAR and MMP-9 protein levels upon lipid raft disruption could be due to downregulation of uPAR and MMP-9 gene expression at earlier time points. Taken together, these results suggest that lipid rafts play an active role in the modulation of uPAR and MMP-9 activities in both MDA-MB-231 and ZR 751 cells. The observed decrease in uPAR and MMP-9 activity upon lipid raft disruption in both cell lines could be very critical to the cellular proliferation of breast carcinoma.

### Lipid raft disruption downregulates the levels of phosphorylated Src, FAK, caveolin, PI3-K, Akt and ERK in MDA-MB-231 and ZR 751 cells

**Figure 5:**
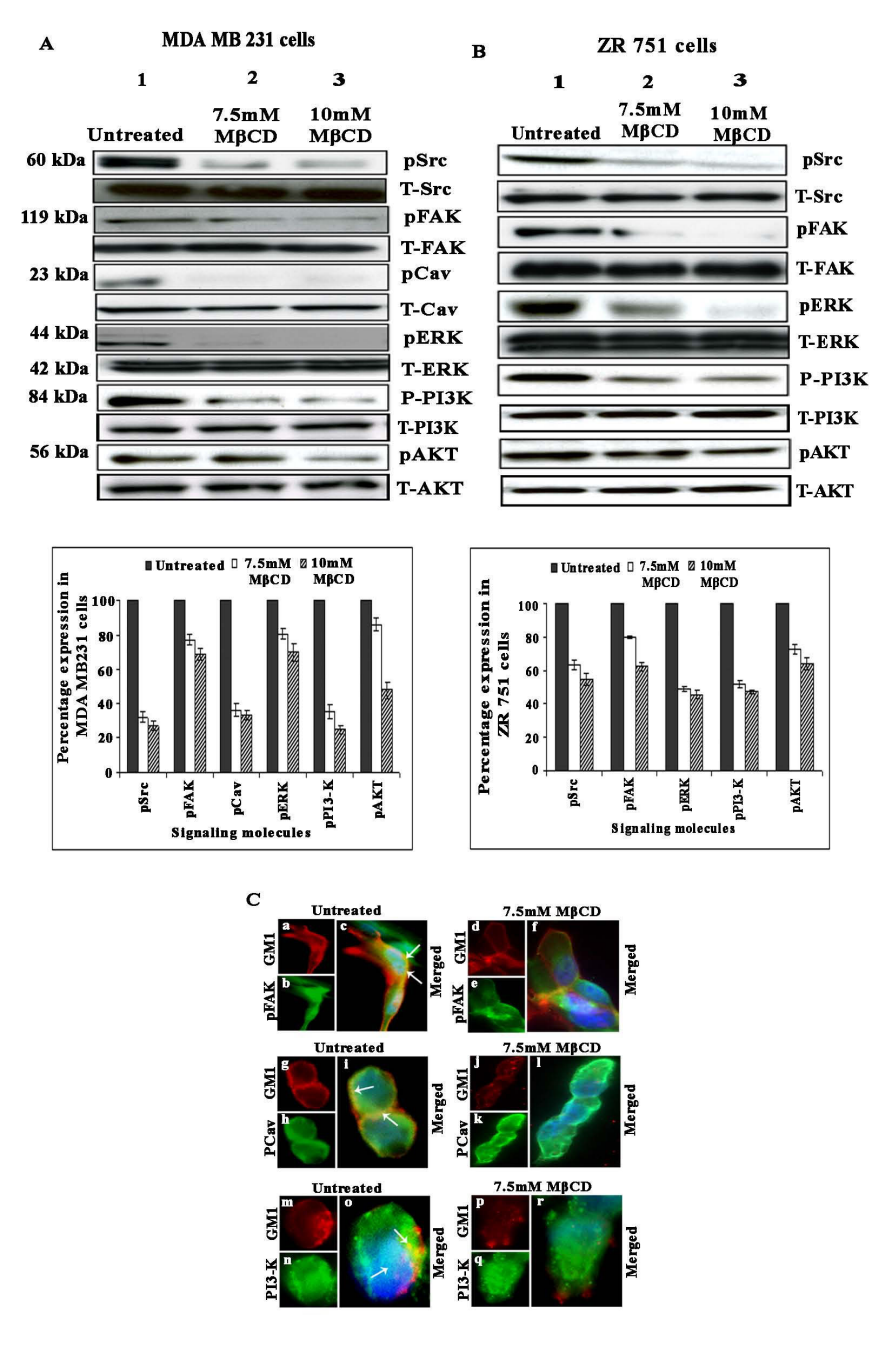
**Lipid raft disruption inhibits the levels of pSrc, pFAK, pCav, pERK, pAkt, pPI3-K, and pAkt in breast carcinoma cell lines**. (A) and (B) MDA-MB-231 and ZR 751 cells left untreated or pretreated with different concentrations of MβCD were subjected to Western blot analysis for total and phosphorylated forms of Src, FAK, Cav, ERK, PI3-K, and Akt. GAPDH was used to verify that similar amounts of protein were loaded in each lane. Densitometric analysis of phosphorylated molecules was done. The result provided is of a representative experiment repeated 3-4 times with concordant results (*p < 0.01, **p < 0.001). (C) MDA-MB-231 cells were left untreated or pretreated with 7.5 mM MβCD for 1 hr at 37°C, washed, labeled with primary antibodies to GM1 by Vybrant lipid raft labeling kit, fixed, permeabilized, incubated with pFAK, pCav and pPI3-K antibodies and appropriate secondary antibodies. The cells were washed, stained with DAPI containing anti-fade agent, and visualized by confocal immunofluorescence microscopy.

Previous studies have shown that Src is associated with lipid raft domains in different cell types [3]. To determine if lipid raft disruption affected Src phosphorylation, lysates prepared from MDA-MB-231 cells before and after treatment with 7.5 and 10 mM MβCD were probed with antibodies against phospho-Src pY416. As expected, Src phosphorylation was high in untreated cells (Figure 5A, upper panel, lane 1). In cells pretreated with MβCD, there was significant downregulation of phospho-Src in both MDA-MB-231 and ZR 751 cells. Previous studies from our lab have shown that uPAR activation in breast carcinoma cells activates Src, FAK, PI3-K and Akt signaling pathways [24]. As most of these signaling molecules are associated with lipid rafts [3], we wanted to check if lipid raft disruption regulates these signaling molecules in both MDA-MB-231 and ZR 751 cells. Western blotting analysis of the lysates revealed higher levels of pFAK, pPI3-K, pAkt, pCav and pERK in untreated MDA-MB-231 and ZR 751 cells (Figures 5A-B). We observed significant reductions in the phosphorylated levels of FAK, Akt, PI3-K, caveolin and ERK in LR-disrupted cells when compared with untreated cells (Figures 5A-B, lanes 2 and 3). Equal loading of total lysates was confirmed by GAPDH. We did not find significant differences in the total levels of Src, FAK, caveolin, ERK and Akt. These results suggest that lipid rafts play a significant role in Src, FAK, PI3-K, Akt and caveolin phosphorylation in MDA-MB-231 and ZR 751 cells.

To further confirm our results, immunofluorescent analysis of untreated and LR-disrupted cells revealed association of FAK, pCav, and PI3-K with the LR marker GM1 in untreated MDA-MB-231 cells (Figure 5C, panels c, i and o) and the absence of this colocalization in MβCD-treated cells (Figure 5C, panels f, l and r). Similar results were observed with pSrc and pERK (data not shown). Overall, these results suggest that lipid rafts play a critical role in Src, FAK, caveolin, ERK and Akt phosphorylation in MDA-MB-231 and ZR 751 cells.

### Lipid raft disruption by cholesterol depletion inhibits migration and invasion of MDA-MB-231 and ZR 751 cells

**Figure 6:**
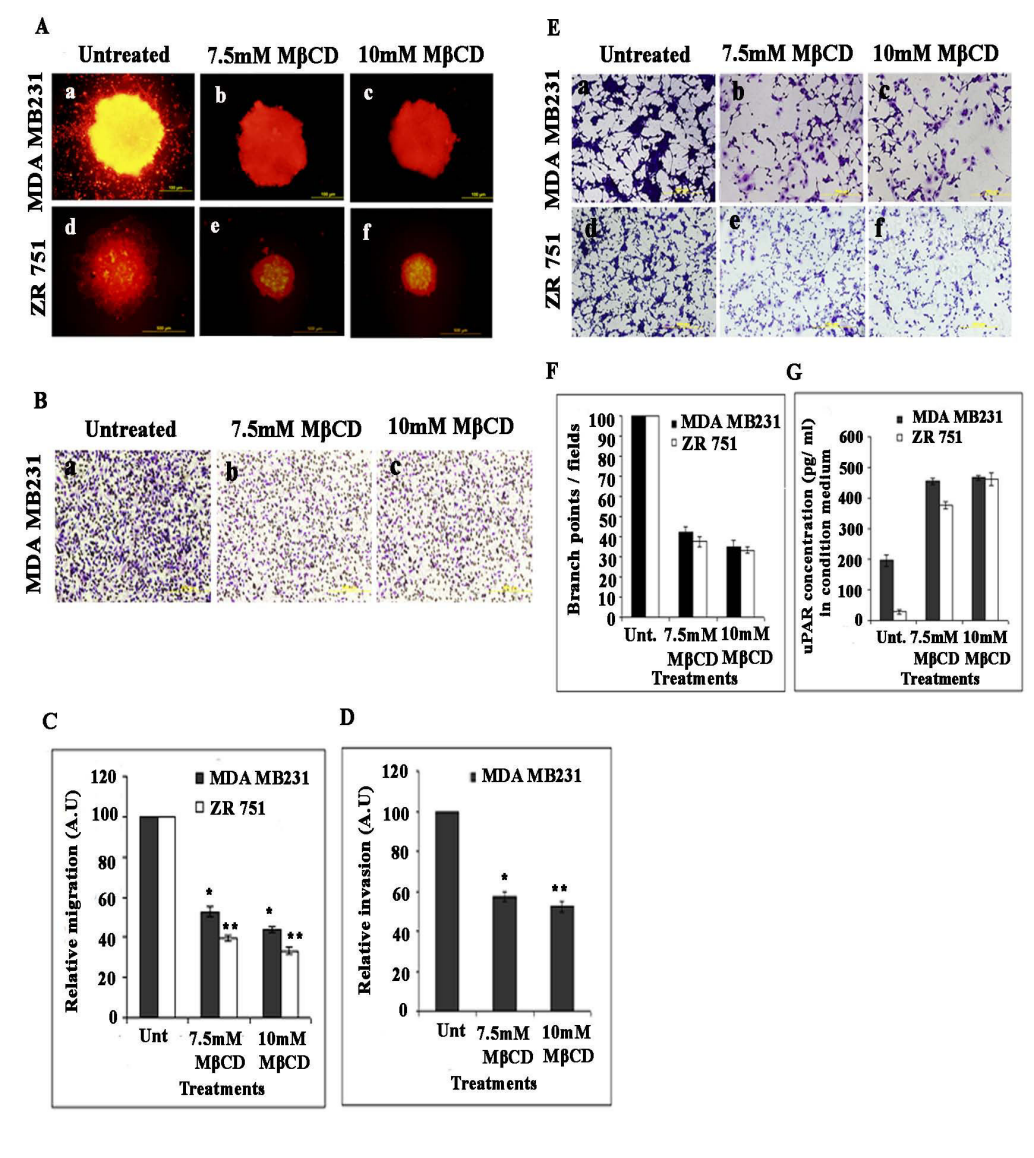
**Lipid raft disruption inhibits migration, invasion and angiogenesis of breast carcinoma cell lines**. (A) Lipid raft disruption inhibits the migration of breast carcinoma cells. Intact MDA-MB-231 and ZR 751 cell spheroids of approximately the same diameter were selected. Spheroids were then transferred in serum-free medium, left untreated or treated with different concentrations of MβCD, and incubated for 48 hrs to allow for migration. Finally, cell migration was observed using a laser scanning microscope. (B) Lipid raft disruption inhibits the invasion of breast carcinoma cells. MDA-MB-231 cells (1 × 10^6^) were left untreated or pretreated with different concentrations of MβCD, washed, and allowed to migrate through Matrigel-coated transwell inserts (8-μM pores) for 24 hrs. The cells that invaded through the Matrigel-coated inserts were stained, counted and photographed under a light microscope. (C) Quantification of relative migration. (D) The percentage of invasion was quantified as described earlier. Values represented are mean ± S.D from three different experiments (**p < 0.001). (E) Lipid raft disruption inhibits angiogenesis of breast carcinoma cells. Conditioned media from MDA-MB-231 and ZR 751 cells, which were left untreated or pretreated with MβCD, were collected at 24 hr. 8 × 10^3 ^human microvascular endothelial cells (HMEC) were cultured in the collected conditioned medium in 48-well plates for 24 hrs. After the incubation period, the medium was removed, and the cells stained with Hema-3 stain and examined under a microscope. (F) Quantification of angiogenesis in endothelial cells that were left untreated or pretreated with MβCD. Values are mean ± S.D. from three different experiments (*p < 0.01, **p < 0.001). (G) Lipid raft disruption increases the secretion of soluble uPAR. MDA-MB-231 and ZR 751 cells were left untreated or pretreated with MβCD, and conditioned medium was collected at the 48 hr. suPAR levels were measured and quantified by ELISA as per the manufacturer's instructions.

Previously in Figures 1 &2 we have shown that LR disruption in MDA-MB-231 and ZR 751 cells inhibited uPAR and MMP-9 activity. Consequently, we evaluated whether LR disruption affects migration and invasion of breast carcinoma cells. Figure 6A shows spheroids, which were left untreated, had a high number of cells that migrated from the spheroids into the surrounding area. However, LR-disrupted cells failed to migrate, which resulted in no migration (Figure 6A, panels a to f). MDA-MB-231 cells were left untreated or pretreated with different concentrations of MβCD and allowed to invade through Matrigel-coated filters. Figure 6B shows the staining of LR-disrupted MDA-MB-231 cells was significantly less than that of the untreated cells. Quantitative analysis of spheroid migration in MDA MB231 and ZR 751 cells before and after treatment with MβCD showed 50-70% reduction in migration (Figure 6C). Quantitative analysis of the cells showed that only 30-40% of the LR-disrupted MDA MB231 cells invaded the Matrigel as compared to the untreated cells (Figure 6D). Taken together, these findings provide strong evidence that by modulating the lipid rafts in breast carcinoma cells, the migration and invasion of MDA-MB-231 cells and ZR 751 cells are significantly inhibited as compared to cells in which the integrity of the lipid rafts were maintained. These results indicate that lipid rafts are critical for the migratory and invasive properties of breast carcinoma cells.

### Lipid raft disruption by cholesterol depletion inhibits angiogenesis of MDA-MB231 and ZR 751 cells

As studies have shown that cholesterol depletion plays a critical role in VEGF signaling [26], we determined whether cholesterol depletion by MβCD plays a role in the angiogeneic potential of breast carcinoma cells. Conditioned medium prepared from the supernatants of MDA-MB-231 and ZR 751 cells that were left untreated or pretreated with different concentrations of MβCD were cultured with human microvascular endothelial cells. We evaluated the tumor conditioned medium-induced vessel formation using an *in vitro *angiogenesis assay. The results showed that conditioned medium prepared from cells that were treated with MβCD significantly reduced the capacity of endothelial cells to form capillary-like structures (Figure 6E, panels b, c, e and f). In contrast, conditioned medium from untreated cells resulted in well-formed capillary-like structures by the 48 hr time point (Figure 6E, panels a and d). The quantification of branch points and number of branches confirmed a significant reduction in the MβCD-treated samples when compared with the untreated cells (Figure 6F). These results suggest that lipid raft disruption downregulates the angiogenic potential of breast carcinoma cells.

### Lipid raft disruption by cholesterol depletion enhances the secretion of soluble uPAR in the conditioned medium of breast carcinoma cells

After having demonstrated the effect of lipid raft disruption on uPAR protein expression in the cell lysates of breast carcinoma cells, we assessed the levels of soluble uPAR in the conditioned medium of LR-disrupted cells at 48 hrs. We used a commercially available uPAR immunoassay detection kit (R&D Systems) and performed the assay as per the manufacturer's instructions. As shown in Figure 6G, we observed a 7-to 8-fold increase in soluble uPAR levels in MDA-MB-231 cells pretreated with the LR-disrupting agent MβCD and a 5-to 6-fold increase in soluble uPAR levels in ZR 751 cells pretreated with MβCD. Overall, these results suggest that lipid raft disruption promotes the removal of uPAR from the plasma membrane, and the released soluble uPAR is secreted for at least 48 hrs in breast carcinoma cells.

### Lipid raft disruption by cholesterol depletion results in uPAR trafficking to the lysosomal pathway in MDA-MB-231 cells

**Figure 7:**
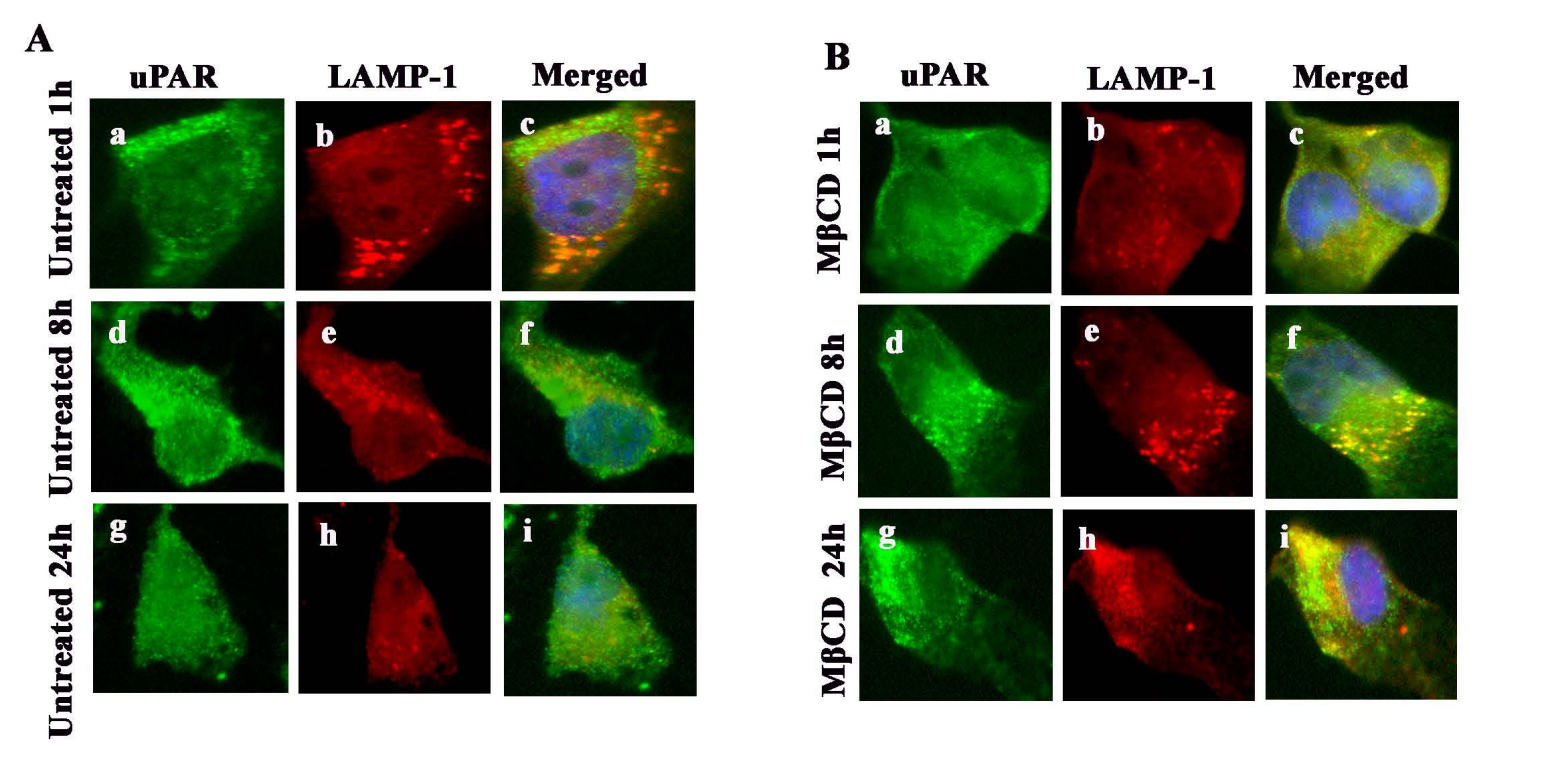
**Time course studies of uPAR before and after lipid raft disruption in MDA-MB-231 cells**. MDA-MB-231 cells were left untreated or pretreated with 7.5 mM MβCD for one hour at 37°C; washed; fixed with 1% paraformaldehyde at 1, 8 and 24 hrs; permeabilized; incubated with primary antibodies against uPAR and LAMP-1 overnight at 4°C; washed; incubated with appropriate Alexa Fluor secondary antibodies; and mounted with an anti-fading agent containing DAPI. The cells were imaged under an Olympus fluoview confocal microscope.

uPAR is known to cycle back to the plasma membrane after it has been endocytosed with LRP, PAI and uPA [27]. To study the fate of uPAR after lipid raft disruption, we carried out trafficking studies of uPAR at different time points using immunofluorescent staining for LAMP-1, a lysosomal marker, and uPAR at 1, 8 and 24 hr time points. In untreated cells, we observed very little colocalization of uPAR with LAMP-1 in MDA-MB-231 cells, suggesting that the majority of uPAR is not targeted to the lysosomal pathway in LR-intact cells (Figure 7A, panels a to i). However, in LR-disrupted cells, we observed significant colocalization of uPAR with LAMP 1 as early as one hour after treatment (Figure 7B, panels a to c) and further increased by 8 hrs (Figure 7B, panels d to f) and 24 hrs (Figure 7B, panels g to i). The results suggest that lipid raft disruption causes the targeting of uPAR to the lysosome, which may contribute significantly to the loss of the uPAR function in breast cancer cells.

### Cholesterol supplementation reverses the downregulated uPAR levels to basal levels in MDA-MB-231 and ZR 751 cells

**Figure 8:**
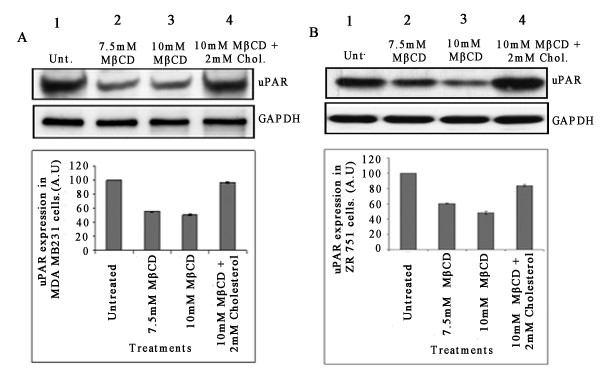
**Supplementation of cholesterol reverses the effect of lipid raft disruption on uPAR levels in MDA-MB-231 and ZR 751 cells**. Serum-starved (A) MDA-MB-231 and (B) ZR 751 cells were left untreated, treated with different concentrations of MβCD, or treated with 10 mM MβCD and supplemented with 2 mM cholesterol. The cells were lysed, and 80 μg of protein from each treatment were subjected to immunoblotting analysis using anti-uPAR and GAPDH antibodies. Similar results were observed in two separate experiments. Abbreviations: uPAR-urokinase plasminogen activator receptor; MMP-matrix metalloproteinase 9, LR - lipid raft, MβCD - Methyl beta cyclo dextrin.

To further examine a possible role for cholesterol in the downregulation of uPAR in MDA-MB-231 and ZR 751 cells, MDA-MB-231 and ZR 751 cells were left untreated, treated with MβCD alone or cholesterol addition after cholesterol depletion. Lysates were prepared and subjected to immunoblotting analysis. MβCD-induced downregulation of uPAR levels in MDA-MB-231 and ZR 751 cells were reversed by the addition of cholesterol as assessed by immunoblotting (Figures 8A-B). These results indicate that cholesterol repletion reverses the MβCD-induced uPAR activity in breast carcinoma cells.

## Discussion

The present study shows that lipid raft disruption by cholesterol depletion could significantly attenuate invasion, migration and angiogenesis in breast carcinoma cells. These functions are mediated by the downregulation of uPAR and MMP-9 and their associated signaling molecules. We have looked at the role of lipid rafts in breast cancer cells by means of cholesterol depletion using MβCD, which has been used in many studies [3,28-30]. Previous studies from our lab [31] have shown that bicistronic constructs against uPAR and MMP-9 have significant inhibitory potential against many types of cancer, particularly breast cancer. As both uPAR and MMP-9 are associated with lipid raft microdomains, we wanted to study the effect of lipid raft disruption in the modulation of uPAR and MMP-9 and their associated signaling molecules. Moreover, as cholesterol is a major component of lipid rafts, depletion of cholesterol has been employed to elucidate the role of LR domains.

Lipid rafts are a source of many signaling proteins including Src, FAK, heterotrimeric G protein subunits and receptor tyrosine kinases [4]. Lipid rafts have been implicated in many types of cancer, and cholesterol accumulation in different types of cancer is well established [32,33]. However, few studies have addressed the effect of lipid raft disruption on the invasive, migratory and angiogenic potential of cancer cells. Studies have shown that uPAR, a GPI anchored protein, is a lipid raft resident protein [34]. Cunningham et al. [7] have shown that the dimeric form of uPAR was particularly enriched in the lipid rafts. They also showed that uPA-induced uPAR cleavage was strongly accelerated in the lipid rafts. In the present study, we observed significant association of uPAR with lipid rafts in both MDA-MB-231 and ZR 751 cells using morphological methods and biochemical isolation of LR fractions in breast carcinoma cells. Whether the lipid rafts of MDA-MB-231 or ZR 751 cells contain the monomeric form or the dimeric form of uPAR was not evaluated in this study.

In the present study, we demonstrate that cholesterol depletion by MβCD lowers the surface expression of uPAR and MMP-9 in breast carcinoma cells. Cholesterol lowering agents like MβCD cause disintegration of lipid rafts and block signaling, thereby suggesting that cholesterol is very critical for LR formation and also its function. Many studies have shown that LR domains are rich in survival-related molecules like PI3-K/Akt and ERK [3]. Our results are in agreement with Li et al. [28] who demonstrated downregulation of Akt in MCF-7 and MDA-MB-231 cells due to elevated levels of rafts in these cells. Studies have also shown that lipid rafts are critical for the function of the Src family of kinases [35,36]. In the present study, we were able to see that LR disruption in MDA-MB-231 cells significantly reduced levels of PI3-K and Src.

Cholesterol depletion resulted in lipid raft internalization associated with FAK downregulation, which probably affected cell adhesion. Cell adhesion is mediated by integrins and lipid raft disruption could affect their interaction, which could have resulted in the loss of cell adhesion. Currently, studies of the association of integrins with LR domains in breast carcinoma cells are under investigation. In our study, we observed that LR disruption by cholesterol depletion reduced levels of uPAR and MMP-9 in breast carcinoma cells as shown by Western blotting and fibrin and gelatin zymography. Previous studies from our lab have shown that uPAR is associated with integrins such as α5β3 and receptors [37]. Moreover, others have shown that the addition of cholesterol increases cell adhesion via ganglioside GM3, which is present in lipid raft domains and α5β1 [38]. Based on our results, it is possible that MβCD treatment alters uPAR and MMP-9 functions and integrin function, which results in decreased cell proliferation and invasion. We are also in the process of determining the role of other signaling molecules like Rho GTPases and lipid rafts in breast carcinoma cells.

Interestingly, we also observed FAK downregulation upon cholesterol depletion in breast carcinoma cells. This is in accordance to a recent study done by Park et al. [39], which demonstrated that cholesterol is critical for FAK-mediated cell survival. The study showed that FAK activation might prevent caveolae internalization, which allows for cell survival signaling like the PI3-K signaling pathway. In our present study, exogenous cholesterol supplementation reversed the levels of uPAR to their basal levels. However, how this phenomenon was induced is still not clear. Our results also showed that lipid raft disruption targets uPAR to the lysosome in MDA-MB-231 cells. As previous studies have shown that cholesterol accumulation is associated with cancer progression [32,33], the targeting of lipid rafts for cancer therapy has been suggested [40,41]. Studies by Zhuang et al. [42] have shown that certain tumors, which are dependent on PI3-K/Akt pathway, are susceptible to cholesterol-targeted drug therapy. Although their contention is largely true, cholesterol depletion also alters uPAR and MMP-9 activity. As shown by immunoassay, the levels of soluble uPAR upon lipid raft disruption in the conditioned medium were increased in cells pretreated with a lipid raft disrupting agent at the 24 hr time point in both cell lines. As determined by zymography, uPA levels in conditioned medium were significantly down regulated upon lipid raft disruption at the 24 hr time point in both cell lines. These results suggest that an intact lipid raft is critical for efficient cancer cell proliferation, and altering its integrity could significantly affect its invasive and migratory properties.

It is well known that several proteases are present in lipid rafts, and they are thought to play a critical role in tumor cell invasion, metastasis and angiogenesis [43-48]. Endothelial cell migration and invasion was also enhanced by rafts enriched with MMP-2/MMP-9 [45,47,48]. In particular, MMP-9 has been shown to play a critical role in tumor invasion, wound healing, metastasis and angiogenesis [10]. Here, we have demonstrated that cholesterol depletion by MβCD reduced MMP-9 protein levels in the lysates of MDA-MB-231 and ZR 751 cells. We were also able to show that uPAR down regulation by cholesterol depletion was inhibited by cholesterol supplementation.

Consistent with our results, cholesterol depletion has been shown to downregulate phospho-Akt and phospho-ERK, which play critical roles in cell survival and induce apoptosis [28]. In the present study, we observed MMP-9 down regulation at the mRNA level at early time points (up to 8 hrs) in MDA-MB-231 and ZR 751 cells. The MMP-9 promoter is known to have several transcription factor-binding motifs, which include AP-1, NF-κB and Sp-1 [49]. Whether LR disruption in MMP-9 inhibition activates AP-1, NF-κB or SP1 will have to be evaluated at a future date. Zhang et al. [14] have shown that when highly metastatic mouse Lewis lung carcinoma cells were treated with MβCD, their invasive capacity was suppressed and MMP-9 activity in the lipid rafts was inhibited. We observed similar results regarding MMP-9 in MDA-MB-231 and ZR 751 cells, which was contradictory to the study by Kim et al. [50] who have shown that in human keratinocytes, cholesterol depletion increased MMP-9 activity. As assessed by *in vitro *angiogenesis assay, cholesterol depletion in breast carcinoma cells affects their angiogenic potential. Studies to understand the molecular mechanisms of the inhibition of angiogenesis and the role of lipid rafts in this process are under progress.

## Conclusions

To summarize, intracellular cholesterol depletion by LR disruption downregulates MMP-9 and uPAR levels and uPAR trafficking in breast carcinoma cells. Our results also show that cholesterol depletion in breast cancer cells downregulates ERK-and JNK-dependent pathways, thereby leading to downregulation of uPAR and MMP-9 expression. Cholesterol supplementation did reverse the change in uPAR expression levels. Therefore, decreasing cholesterol levels by LR disruption could provide a good strategy to prevent MMP-9 and uPAR-mediated degradation of the extracellular matrix in breast carcinoma.

## List of Abbreviations

UPAR: urokinase plasminogen activator receptor; MMP-9: matrix metalloproteinase 9; LR: lipid raft; SRC: sarcoma; FAK: focal adhesion kinase; ERK: extracellular regulated kinase; PI3-K: phosphotidyl inositide 3 kinase; CAV: caveolin; MβCD: methyl beta cyclodextrin; GAPDH: glyceraldehyde phosphate 3 dehydrogenase; NAC: N-acetyl cysteine; GM1: monosialotetrahexosyl ganglioside; and LAMP-1: lysosome associated membrane protein 1.

## Competing interests

The authors declare that they have no competing interests.

## Authors' contributions

HR, PKS, RRM, and CG carried out the experiments described in the study. HR wrote the manuscript. HR, JSR conceived, reviewed and analyzed the data. JSR and NE Contributed reagents/materials/analysis tools. All authors read and approved the final manuscript.

## Pre-publication history

The pre-publication history for this paper can be accessed here:

http://www.biomedcentral.com/1471-2407/10/647/prepub
